# A novel approach for the treatment of AML, through GHRH antagonism: MIA-602

**DOI:** 10.1007/s11154-024-09917-6

**Published:** 2024-10-17

**Authors:** Joel Costoya, Simonetta I. Gaumond, Ravinder S. Chale, Andrew V. Schally, Joaquin J. Jimenez

**Affiliations:** 1 Dr. Phillip Frost Department of Dermatology and Cutaneous Surgery, Miller School of Medicine, University of Miami, Miami, FL USA; 2 Department of Biochemistry and Molecular Biology, Miller School of Medicine, University of Miami, Miami, FL USA; 3 Division of Endocrinology, Department of Medicine, Miller School of Medicine, University of Miami, Miami, FL USA; 4 Division of Hematology & Oncology, Department of Medicine, Miller School of Medicine, University of Miami, Miami, FL USA; 5 Department of Pathology, Miller School of Medicine, University of Miami, Miami, FL USA; 6 Endocrine, Polypeptide and Cancer Institute, Veterans Affairs Medical Center, Miami, FL USA; 7 Department of Medicine, Sylvester Comprehensive Cancer Center, Miller School of Medicine, University of Miami, Miami, FL USA; 8 University of Miami Miller School of Medicine, Miami, FL USA

**Keywords:** Targeted therapy, Chemotherapy, AML, MIA-602, Drug-resistance, GHRH antagonism

## Abstract

Acute myeloid leukemia (AML) is the most aggressive and prevalent form of leukemia in adults. The gold-standard intervention revolves around the use of chemotherapy, and in some cases hematopoietic stem cell transplantation. Drug resistance is a frequent complication resulting from treatment, as it stands there are limited clinical measures available for refractory AML besides palliative care. The goal of this review is to renew interest in a novel targeted hormone therapy in the treatment of AML utilizing growth hormone-releasing hormone (GHRH) antagonism, given it may provide a potential solution for current barriers to achieving complete remission post-therapy. Recapitulating pre-clinical evidence, GHRH antagonists (GHRH-Ant) have significant anti-cancer activity across experimental human AML cell lines in vitro and in vivo and demonstrate significant inhibition of cancer in drug resistant analogs of leukemic cell lines as well. GHRH-Ant act in manners that are orthogonal to anthracyclines and when administered in combination synergize to produce a more potent anti-neoplastic effect. Considering the adversities associated with standard AML therapies and the developing issue of drug resistance, MIA-602 represents a novel approach worth further investigation.

## Introduction

Hematopoiesis occurs as higher potency hematopoietic stem cells self-renew and differentiate towards multipotent progenitors, eventually becoming lineage-committed progenitors of the myeloid and lymphoid families. The common myeloid and lymphoid progenitors will undergo subsequent maturation leading to the formation of granulocytes, macrophages, platelets, erythrocytes, and lymphocytes [1]. AML is a malignancy of hematopoietic origin characterized by recurrent alterations in chromosomal, molecular, and epigenetic programs responsible for the differentiation, survival, and proliferation of myeloid progenitor cells [2]. A distinguishing feature of AML is a myeloblast count greater than or equal to 20% of cells found in the bone marrow, or peripheral blood [3]. Due to this accumulation of immature leukemic cells, the bone marrow becomes dysfunctional, often resulting in effects associated with cytopenias. Constitutional symptoms of AML include anergy, malaise, and fever, amongst others, followed by recurrent infections and/or other hemorrhagic complications.

Amongst adult populations, AML stands as the most prevalent form of leukemia and has the poorest prognosis, at a 31.7% five-year relative survival rate [1]. Conventional treatment paradigms for patients diagnosed with AML involve the use of intense chemotherapeutic intervention and may ultimately include allogeneic hematopoietic stem cell transplantation [4]. Patients unable to withstand the aforementioned strategies may opt for less intense therapies such as DNA hypomethylating agents, low-dose Cytarabine, or best supportive care. Advancements in genome sequencing, cytogenetic analysis, morphological classification, and immunophenotyping, paired with our evolving understanding of leukemic pathobiology has allowed physicians to further refine diagnosis, as well as prognosis in AML. Concomitantly, these developments also serve as a guide for therapeutic intervention and novel targeted therapies [1].

In recent decades, the treatment of acute promyelocytic leukemia (APL), a subtype of AML, underscores the impact of targeted therapies and translation of clinical research. First described in 1957, distinguished by a promyelocytic morphology and rapidly fatal course due to hemorrhagic complications as a result of fibrinolysis, APL accounts for approximately 10% of all AML. As it stands, if diagnosed early, APL has the most favorable prognosis of all sub-types of AML [5]. APL’s hallmark trait is a balanced chromosomal translocation t(15:17) (q24.1;21.1), leading to the expression of a fusion protein known as Promyelocytic leukemia gene (PML)-retinoic acid receptor-alpha (RARA). PML-RARA is an aberrant retinoic acid receptor, which through its PML moiety recruits corepressors for the transcription of genes associated with retinoic acid response elements (RAREs) whilst preventing coactivator recruitment [5].

Administration of all-*trans* retinoic acid (ATRA) causes the degradation of PML-RARA protein, thus preventing the repression of retinoic acid activated genes, allowing for coactivator recruitment and subsequent transcription of RAREs. The response seen is differentiation of promyelocytes and a halt in proliferation both in vitro and in vivo [5]. Clinically ATRA should be given immediately upon suspicion of an APL diagnosis, as it drastically attenuates risk of severe bleeding events due to coagulopathies [1, 5]. Addressing resistance to retinoic acid became an issue shortly after incorporating ATRA into therapeutic regimes, partially alleviated by the recognition of arsenic trioxide (ATO) compounds. ATO targets the PML moiety of the PML-RARA fusion protein inducing its degradation, similarly to ATRA, it causes differentiation of APL cells and blocks their proliferation [1, 5]. Despite their relative success and proof of efficacy, long-term disease-free survival (DFS) is still a concern due to inevitable relapse seen in up to 20% of APL patients, furthermore increased metabolism and tolerance of ATRA and ATO drugs creates a significant hurdle in disease remission. The need for circumventing this is especially true in high-risk patients (WBC > 10,000 cells/µL), where combination of ATRA and ATO might not be sufficient, requiring the use of cytotoxic agents. High-risk patients are more likely to experience relapse following anthracycline/ATRA regimens and develop resistance, requiring ATO implementation, still a subset of patients remain unresponsive [1]. At present, much like in the earlier described treatments of AML, acquired drug resistance remains at the forefront of obstacles to complete remission (CR) and necessitates the need for novel targeted therapies which can utilize alternate pathways in their mechanism of action or improve upon conventional treatment practices [5].

Targeted hormone medications have emerged into the treatment of various kinds of cancer as an adjuvant therapy subsequent to primary interventions such as surgery, radiotherapy, or chemotherapy. GHRH is a neuropeptide secreted by the hypothalamus, which acts on the pituitary gland to stimulate the synthesis and release of growth hormone (GH). Originally GHRH was only thought to be responsible for the pituitary GH/hepatic insulin-like growth factor 1 (IGF-1) axis, and thus regulation of physiological levels of GH and IGF-1. The discovery of GHRH peptides and GHRH receptor (GHRH-R) in several extrahypothalamic tissues alluded to a potential independent role than that of GH stimulation [6–9].

For many years, a wide diversity of cancers were known to produce GHRH and express receptors for it, yet only recently was it proposed to function as an autocrine growth factor for neoplasms [10–12]. Surgical specimens of human prostate, breast, endometrial, and ovarian cancers were identified to contain GHRH mRNA [13], and a variety of human cancer lines and tumor samples express mRNAs encoding four possible splice variants (SVs) of GHRH-Rs with high-affinity binding sites for GHRH and its analogs [14]. Synthetic GHRH-Ant were then produced in order to develop a class of anti-cancer agents, demonstrating inhibition of human tumor growth in vitro and in vivo against renal [15], bone [16], breast [17, 18], pancreatic [19], prostatic [20, 21], colorectal [22], and lung cancers [23]. GHRH peptide was also localized to immune cells, such as monocytes, T cells, and B cells [24, 25] leading to the first attempt of using GHRH-R antagonism as a means of treating lymphoma. Experiments modeling non-Hodgkin’s Lymphoma in cell lines and tumors xenografted into nude mice showed remarkable efficacy for GHRH antagonism therapy, further cementing the assumption of tumor growth inhibition as a direct effect of GHRH antagonism on malignant lymphoma cells and showcasing a novel means of achieving remission [26].

Expanding upon these findings, the use of GHRH antagonism, particularly with MIA-602, was successfully applied in vitro and in vivo to both AML and APL models, featuring a chemotherapy-selected derivative of the K562 cell line incubated against increasing concentrations of Doxorubicin, and a ATRA + ATO resistant NB4 subline [27–31]. In pre-clinical studies, MIA-602 has shown great potential as an anti-neoplastic agent against cancers of the prostate [32], breast [17], ovary [33], endometrium [34], stomach [35], and glioblastoma [36] whilst maintaining weaker endocrine inhibitory activity than other GHRH-Ant [34]. The accumulated evidence indicates a susceptibility in AML, to MIA-602 mediated GHRH-R antagonism in a significant time- and dose-dependent manner by a mechanism of action that is distinct to commonly used drug-based targeted therapy regimens [27–31]. MIA-602 is thought to manifest its anti-neoplastic effects partially by systemic inhibition of the pituitary GH/hepatic IGF-1 axis, albeit the blocking of autocrine and paracrine activity from onco-derived GHRH, and GHRH-R downregulation resulting from direct binding of GHRH-Ants to tumoral GHRH-Rs seems responsible for most of its biological interactions [34]. The result of GHRH-Ant binding activity includes but is not limited to modulation of PI3K/Akt and ERK1/2 signaling, activation of pro-apoptotic mechanisms, regulation of STAT3/NF-κB and TNF-α inflammatory pathways, and remodeling of cellular motility networks involving E-cadherin and caveolin-1 [31]. Considering the adverse effects associated with the use of chemotherapies and differentiating agents in the treatment of AML, a non-cytotoxic agent like MIA-602 [34], represents a new approach in clinical therapies that might avoid the unwanted side effects of current medications and successfully addresses acquired drug-resistance.

The purpose of this paper is to review pre-clinical evidence surrounding the use of MIA-602 and discuss the potential for new targeted hormone therapies based on GHRH-R antagonism in the treatment of AML. Given its outstanding success and profound clinical implications, it is reasonable to advocate for the development of randomized controlled trials and cohort studies on possible GHRH-Ant based therapies. Further evaluation of the safety and efficacy, as well as long-term monitoring of effects from MIA-602 as a mono- or adjuvant therapy in patients is lacking, and certainly needed. Renewed interest in this approach could lead to significant improvements in the field of AML treatment as a whole.

## A new class of anti-neoplastic agents

Evidence of a growth factor responsible for the release of GH, from hypothalamic extracts, was first demonstrated during the 1960’s. Two decades later, the structure of GHRH was isolated in its 40 and 44- amino acid forms and characterized from ectopic productions of pancreatic tumors related to acromegaly [37, 38]. This neuroendocrine hormone became subsequently identified as the same agent capable of inducing the secretion of GH from the pituitary, found in both the animal and human hypothalamus. Growth hormone-releasing hormone is well conserved across many species and belongs to the same family of peptide hormones, as secretin, vasoactive intestinal polypeptide (VIP) and pituitary adenylate cyclase-activating polypeptide (PACAP) [39]. Despite its discovery in tumoral productions hinting at its potential in the field of oncology, GHRH’s posited role in the development of cancer remained largely unexplored until the last few decades [40].

After its production in the hypothalamus, GHRH binds to GHRH-R found on somatotrophs in the anterior pituitary gland stimulating cAMP, Ca^2+^-calmodulin, and other secondary messengers to induce GH synthesis and secretion [7]. This is often working in opposition to somatostatin [33]. GH’s bioactivity leads to growth of virtually all tissues throughout the body, this is accomplished by promoting fatty acid catabolism and stimulating protein synthesis, providing the necessary energy and building blocks needed to sustain growth [41, 42]. It is well understood that in the liver, the binding of GH to GH receptors leads to the production of IGF-1, which is also known to be a potent mitogenic factor and contributes to the growth of a plethora of cell types, cancers included [43]. GHRH’s contribution to carcinogenesis was speculated to have to do with its role in the pituitary GH/hepatic IGF-1 axis and its regulation of physiological levels of GH and IGF-1. Indeed, GHRH-Rs in the body are localized mostly on somatotrophs of the anterior pituitary, due to tissue-specific expression of the transcription factor Pit-1 [39]. Additionally, GH and IGF-1 were found to be implicated in the metastatic epithelial-to-mesenchymal transition (EMT), malignant transformation, and growth of various neoplasms [43].

A need for clinical implementation of a GHRH-Ant arose from the inability of somatostatin analogs to lower GH and IGF-1 levels sufficiently enough to inhibit the growth and progression of IGF-dependent cancers, as well as a growing body of evidence painting GHRH as a pleiotropic autocrine growth factor [44–46], apart from its role in the pituitary [6, 7]. Predominantly, GHRH is produced in the hypothalamus, yet many peripheral tissues as well as several cancer lines and tumors have been shown to express GHRH [7, 23]. This has been corroborated by literature illustrating mRNA for GHRH peptides, as well as mRNA for the GHRH-R and up to four different splice variants, SV1 being the most common form, both in vitro and in vivo across multiple cancer models [47]. The presence of bioactive GHRH-Rs in numerous surgical carcinomas has also been confirmed by immunocytochemical localization [48], and treatment of over 60 human cancer cell lines with GHRH-Ants have yielded decreases in cell proliferation, survival, and motility, overall inhibited the progression of cancer [27, 49].

The shortest polypeptide sequence of GHRH that retains its intrinsic biological activity is 29 amino acids long termed GHRH(1–29)NH_2_ [43]. Based on this knowledge, directed evolution and rational design methodologies have served as a platform for engineering novel antagonistic and agonistic peptide analogs of GHRH. Robberecht reported the first of the GHRH-Ants, beginning with the replacement of alanine for D-arginine at position 2 of GHRH(1–29)NH_2_ [50], since then “MZ” [51] and the ensuing “JV” [52] families of GHRH-Ants have been produced, amongst others, by utilizing non-natural amino acid substitutions, amide bond conversions, additions of conformational constraints, and incorporation of polymeric tags. As shown in Table 1, many groups have managed to synthesize analogs capable of selectively targeting malignant cells with enhanced receptor binding affinity, increased bioavailability, heightened resistance to proteolytic degradation and renal clearance pathways, whilst exhibiting improved anti-neoplastic activity and mitigating unwanted side effects [34, 43, 53, 54]. The “MIA” species of GHRH-Ants are the result of our latest efforts in synthesizing antagonistic GHRH analogs, with MIA-602 representing a novel therapeutic approach in the treatment of refractory AML [27, 29, 31].

**Table 1:** Development of GHRH antagonists

GHRH Antagonist Class	Relative Binding Affinity vs. GHRH(1–29)NH_2_	Pharmacodynamic trends	Citation
GHRH (1–29)NH_2_ standard antagonist	1	With the evolution of GHRH-Ants we see an increasing ability to inhibit GH release, enhancements of anti-neoplastic activity, increased binding affinity to GHRH-R and SVs, prolonged bioavailability and enhanced resistance to proteolysis. With the newest “MIA” series, the most anti-neoplastic activity was seen whilst exhibiting decreased endocrine inhibitory activity and a lower binding affinity.	[34]
“MZ-4-71” “MZ-5-156”	26.18, 48.8		[8, 51]
“JV-1-36” “JV-1-38”	62,57		[52]
“MZ-J-7-138”	9.8		[32]
“JMR-132”	2.6		[34]
“MIA-602”	30.8		[34]

This table was assembled based on sources mentioned in this review

**Fig. 1:**
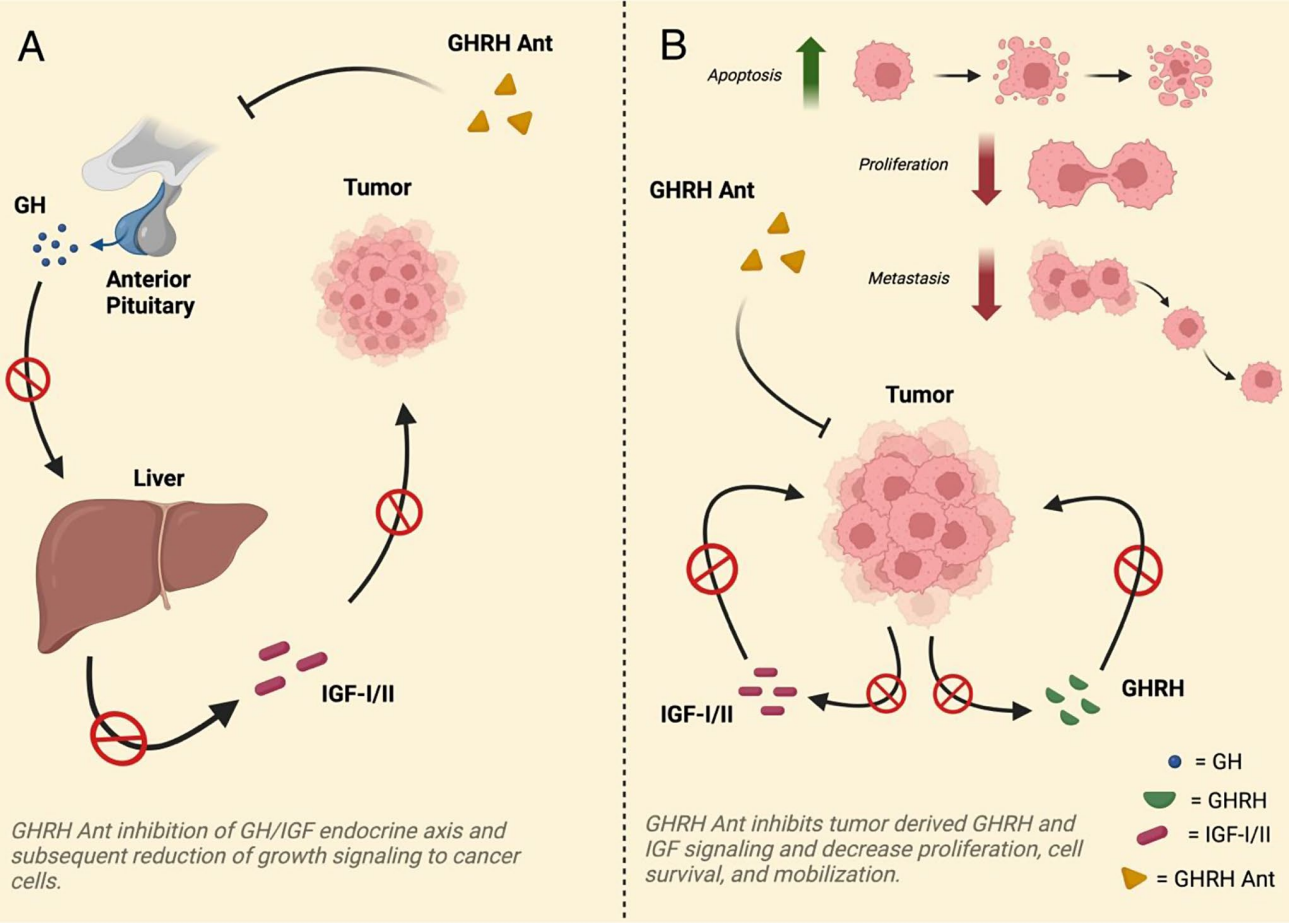
Schematic representation of the pathways influenced by GHRH antagonism to achieve inhibition of a cancer’s ability to rapidly divide, avoid apoptosis, metastasis, and overall progression. Panel **A**: Shows down regulation of the pituitary GH / hepatic IGF endocrine axis. First there is inhibition of GHRH-stimulated pituitary GH release, this leads to the subsequent suppression of GH-stimulated hepatic IGF production, which in turn decreases IGF growth signaling resulting in attenuation of cancer progression. Panel **B**: Shows direct inhibition of autocrine/paracrine stimulatory loops from both tumor derived GHRH and IGF. GHRH-Ants inhibit autocrine/paracrine tumoral production of GHRH and IGF, thus limiting available growth signaling, resulting in decreased cell proliferation, cell survival, and cellular motility. *Created in BioRender. Costoya*, *J. (2024) BioRender.com/g87a784*

The mechanism of action of MIA-602, like other GHRH-Ants, remains to be fully.

elucidated. For the purposes of fighting cancer, in vivo observations thus far have concluded that GHRH-R antagonism works by competitive inhibition, leading to the suppression of pituitary and peripheral GHRH-Rs alike [34]. Consequently, this decreases bioavailable IGF-1, inhibiting the growth of IGF-1-dependent cancers through endocrine, autocrine and/or paracrine signaling. Notably a number of in-vitro studies, where the effects of the pituitary GH/hepatic IGF-1 axis are eliminated, have demonstrated that direct tumoral GHRH-R binding of antagonistic GHRH analogs can work independent of IGF to inhibit carcinogenesis. GHRH-Ant binding activity seemingly blocks autocrine/paracrine mechanisms of GHRH and brings about decreases in cellular survival, inhibits proliferation, and rewires cell motility networks, resulting in decreased potential for metastatic transition [43, 55], as shown in Fig. 1. Across the 20 varieties of cancer that GHRH-Ants have been experimented with, PI3K/Akt/mTOR and Raf/MEK/ERK 1/2 signaling is commonly inhibited [27], as well as the production of cAMP [56]. Other documented effects include a reduction in telomerase activity [36], attenuation of the EMT [55], modulation of inflammatory cytokines [17], increases in apoptosis [49], down regulation of STAT3/Pak1 [35], a lessening of the expression of aberrant tumor suppressor genes and oncogenes [17], and interference of angiogenic factors [46, 57], as shown in Table 2. In addition to this, GHRH-Ants offer favorable toxicity profiles since they are non-cytotoxic to non-neoplastic tissues [34], making them a viable candidate for incorporation into current therapeutic regimes. Interestingly newer iterations of GHRH-Ants, including MIA-602, despite having lower inhibitory activity for the GH/IGF-1 endocrine axis, are showing dramatically increased anti-neoplastic potency and pointing to the importance of direct binding of GHRH-Ants to GHRH-R variants [34].

GHRH-Ants have been tested in cancers of varying origin and pathology, yet their potential in the treatment of AML, until recently, had been largely unexplored [27]. Previous discoveries featuring the presence of GHRH mRNA and peptides in immune cells, coupled with successful in-vitro and in-vivo attempts against experimental models of non-Hodgkin’s Lymphoma using earlier GHRH-Ants [26] prompted the investigation of newer, more potent GHRH-Ants for treating AML, particularly MIA-602 [27].

**Table 2:** Documented use of GHRH-Ants against hematological malignancies: their biochemical interactions and observed effects

Cancer Model / GHRH-Ant Used	Biochemical Pathway Interactions	Effects of Treatment	Citation
AML (K562, THP-1, KG-1α, U-937) MIA-602	MEK/ERK 1/2 PI3K/Akt	Anti-proliferative, Anti-tumorigenic, Apoptotic, Decreased Cell Survival, Inhibited ERK and AKT signaling	[27–31]
AML doxorubicin-resistant (K562, U-937, KG-1α) MIA-602	MEK/ERK 1/2 PI3K/Akt	Anti-proliferative, Anti-tumorigenic, Apoptotic, Decreased Cell Survival, Inhibited ERK and AKT signaling	[31]
APL (NB4) MIA-602	MEK/ERK 1/2 PI3K/Akt	Anti-proliferative, Anti-tumorigenic, Apoptotic, Decreased Cell Survival, Inhibited ERK and AKT signaling	[28, 29]
APL doxorubicin-resistant (NB4) MIA-602	MEK/ERK 1/2 PI3K/Akt	Anti-proliferative, Anti-tumorigenic, Apoptotic, Decreased Cell Survival, Inhibited ERK and AKT signaling	[28, 29]
Non-Hodgkin’s Lymphoma (RT, HT) MZ-5-156 MZ-J-7-138	IGF-I, bFGF	Anti-tumorigenic, Anti-proliferative, Apoptotic, Decreased serum IGF-I and IGF-1 mRNA expression, Decreased bFGF production	[26]

This table was assembled based on sources mentioned in this review

## MIA-602 in the treatment of acute myeloid leukemia

Jimenez et al. first confirmed the existence of GHRH-Rs and expression of GHRH in three human AML cell lines: K-562, THP-1, and a subclone of KG-1 named KG-1α. In vitro incubation of aforementioned cancer lines with MIA-602, produced a significant dose- and time- dependent reduction in cell proliferation and induced apoptotic genes increasing overall apoptosis. Significant tumor growth inhibition was also seen in vivo with xenografted tumors in athymic nude mice of the GHRH-R positive cell lines. 9/9 primary cell samples from AML patients were confirmed positive for the expression of GHRH-R through western blotting making the application of GHRH-R antagonism an attractive therapeutic modality. The mechanism of action for MIA-602 in these leukemic models was thought to occur through apoptosis stimulation and suppression of Akt signaling [27].

MIA-602 anti-hormonal therapy was then expanded to the treatment of APL, modeled with promyelocytic leukemia cell line NB4, and was evaluated against an experimentally generated ATRA and ATO resistant clone of NB4 (NB4-RAA) by Jimenez et al. GHRH-Rs were found to be present in the NB4 promyelocytic cell line and were retained in NB4-RAA cells despite incubation against increasing concentrations of ATRA and ATO over the course of twelve months. A significant and comparable decrease in cell proliferation and cell viability, was seen in vitro amongst NB4 and NB4-RAA cells following exposure to MIA-602 in a time- and dose- dependent manner. The same was discovered in athymic nude mice xenografted with NB4 and NB4-RAA tumors displaying significantly reduced tumor volumes and prolonged doubling times. Macroscopic examination of mice tissues was unremarkable, and there were no signs of abnormal change in organ/body mass. MIA-602 was shown to elicit an apoptotic response that is distinct from that caused by retinoic acid and arsenic derivatives with a favorable toxicity profile [34]. Furthermore, synergy of anti-neoplastic effects was observed in vitro when administering both doxorubicin and MIA-602 to K-562 leukemia cells [5, 28, 29].

Lastly, human AML cell lines KG-1α, U-937, and K-562 were selected to create doxorubicin resistant clones over a twelve-month duration. KG-1α, U-937, and K-562 and their doxorubicin resistant sublines were then challenged with increasing concentrations of either doxorubicin, MIA-602, or a combination of doxorubicin and MIA-602. From these endeavors U-937 was identified as another AML model susceptible to MIA-602. Similar to earlier studies, all cell lines experienced a significant time- and dose- dependent reduction in cell viability in response to MIA-602 treatment. Again, synergistic effects in cancer cell inhibition were observed when MIA-602 was applied in combination with doxorubicin, this time against three different cell lines. K-562 doxorubicin resistant cells were then xenografted into athymic nude mice and treatment with MIA-602 showed a significant reduction in average tumor volume against the placebo group in vivo. No sequelae of cytotoxic effects were seen in the mice organs when examining gross anatomy, the native organs and body mass all presented normally. Evidently, MIA-602’s mechanism of action is independent of pathways associated with acquired drug tolerance, a common side-effect of traditional chemotherapeutics used in the treatment of AML, such as doxorubicin [58], and is able to circumvent natural oncogenic defenses in a significant and efficacious manner [30, 31]. The preclinical evidence surrounding this novel work in relation to MIA-602 is representative for GHRH antagonism as a viable candidate for mono- or adjuvant targeted hormone therapies adapted into conventional AML treatment regimens. Further investigation could have deep implications for clinical success, especially in the case of resistant leukemic subtypes.

## Conclusion and future perspectives

GHRH-R antagonism as a novel targeted hormone therapy has promising pre-clinical evidence, warranting efforts towards the development of GHRH-R antagonists as a new class of medications. The therapy would likely bear semblance to other established hormone analogs, currently, there are none available based off GHRH. With respect to AML therapy, MIA-602 has demonstrated GHRH-R antagonism to be quite successful in addressing acquired drug resistance, a primary hurdle against total remission of AML. There are additional potential benefits to these therapies, GHRH-Ants seemingly work in unison with already established drugs to potentiate an anti-neoplastic effect while minimizing new toxicities in non-cancerous tissues. Overall, their clinical significance has been showcased across the literature.

As it stands, GHRH-Ants will likely require continued development as they navigate randomized control trials evaluating their safety and effectiveness, as well as long-term cohort studies to monitor chronic effects associated with GHRH antagonism. Considering adverse effects resulting from treatment complications and the limited options for therapy in refractory AML, continued investment into the production of more potent, effective GHRH-Ants is quite reasonable. The purpose of this review has been to generate interest in a novel targeted hormone therapy, particularly the use of GHRH antagonism in the treatment of AML.

## Data Availability

No datasets were generated or analysed during the current study.

## Abbreviations

AML: Acute Myeloid Leukemia
APL: Acute Promyelocytic Leukemia
PML: Promyelocytic Leukemia gene
RARA: Retinoic acid receptor-alpha
RARE: Retinoic acid response element
ATRA: All-trans retinoic acid
ATO: Arsenic trioxide
DFS: Disease-free Survival
WBC: White blood cell
CR: Complete Remission
GHRH: Growth hormone-releasing hormone
GH: Growth hormone
IGF-I: Insulin-like growth factor 1
SVs: Splice variants
VIP: Vasoactive intestinal polypeptide
PCAP: Pituitary adenylate cyclase-activating polypeptide
EMT: Epithelial-to-mesenchymal transition
GHRH-R: Growth hormone-releasing hormone receptor
